# Can a Peripheral Blood Marker for Airway Neutrophilia Be Identified in Children with Bronchiectasis?

**DOI:** 10.3390/children13020174

**Published:** 2026-01-27

**Authors:** Hendrik Willem Wiltingh, Julie Marchant, Anne Chang, Vikas Goyal

**Affiliations:** 1 Department of Respiratory and Sleep Medicine, Queensland Children’s Hospital, Brisbane, QLD 4101, Australia; hinse.wiltingh@zrt.nl (H.W.W.); jm.marchant@qut.edu.au (J.M.); anne.chang@menzies.edu.au (A.C.); 2 Australian Centre for Health Services Innovation, Queensland University of Technology, Brisbane, QLD 4000, Australia; 3 NHMRC Centre for Research Excellence in Paediatric Bronchiectasis (AusBREATHE), Child and Maternal Health Division, Menzies School of Health Research, Charles Darwin University, Darwin, NT 0811, Australia; 4 Department of Pediatrics, Gold Coast Health, Gold Coast, QLD 4215, Australia

**Keywords:** bronchiectasis, children, inflammatory markers

## Abstract

**Highlights:**

**What are the main findings?**

- Airway neutrophilia is common in children with bronchiectasis.
- Airway neutrophilia is associated with the presence of *Haemophilus influenzae*, *Streptococcus pneumoniae,* and Adenovirus, but there are no reliable peripheral blood markers for airway neutrophilia.

**What are the implications of the main findings?**

- Clinicians should continue to base assessment of airway inflammation in paediatric bronchiectasis on clinical features and lower airway sampling where indicated, rather than peripheral blood inflammatory ratios.
- CRP may provide supportive information but is not disease specific.

**Abstract:**

**Background:** Airway bacterial infection and inflammation are often present in children with bronchiectasis. Systemic inflammation has also been reported. Currently, there are no data on the association between systemic inflammatory markers with airway pathogens or neutrophilia in children with bronchiectasis. We aimed to define the bronchoalveolar lavage (BAL) pathogens (bacteria and viruses), and cytology in children with bronchiectasis and to explore any association between peripheral inflammatory markers and airway neutrophilia. **Methods**: Participants numbering 402, aged <18 years, with peripheral blood and BAL results within 3 months of diagnosis of bronchiectasis were included. Blood and BAL results were reviewed and analysed for possible associations. **Results:** Of 355 children (88.31%), cultured bacteria from BAL and *Haemophilus influenzae* (n = 185) were the most frequent. A virus was identified in 131 (32.59%). Adenovirus (n = 69) was most common. Children numbering 279 (69.40%) had airway neutrophilia (neutrophils > 15%) which was associated with the presence of *H. influenzae* (OR 2.03 95% CI 1.31–3.15, *p* = 0.002), *S. pneumonia* 2.41 (95% CI 1.36–4.29, *p* = 0.003), and Adenovirus (OR 2.06 95% CI 1.06–4.04, *p* = 0.033). Airway neutrophilia was associated with raised CRP (OR 2.26 95% CI 1.14–4.49, *p* = 0.019), but there were no other systemic inflammatory markers including monocyte/lymphocyte ratio, neutrophil/lymphocyte ratio, platelet/lymphocyte ratio, and platelet/mean platelet volume ratio. **Conclusions:** In children, there is an association between airway neutrophilia and raised CRP in bronchiectasis, but not with other peripheral inflammatory markers. There is a need to identify non-invasive inflammatory markers in children with bronchiectasis.

## 1. Introduction

Bronchiectasis is a chronic respiratory condition characterized by a chronic wet/productive cough with frequent respiratory exacerbations diagnosed by abnormally dilated bronchi in the chest computerized tomography (CT) scan [1]. Bronchiectasis is the outcome of a complex interplay between genetics, the host’s environment, airway pathogens, and inflammation leading to airway obstruction, bronchial wall damage, and later abnormal airway dilatation [2]. This interplay has been termed a vicious vortex [3,4]. While predominantly an airway disease, systemic inflammation was shown to be present both in the steady state phase and during exacerbation [5,6]; however, these studies were predominantly in adults with bronchiectasis. In addition, most of the systemic inflammatory markers known to be elevated in adults with bronchiectasis are cytokines and chemokines (IL-8, IL-6, and circulating adhesion molecules ICAM-1, VCAM-1 and E-selectin) [7] that are not measured in routine clinical labs and therefore have limited clinical utility. The ideal peripheral biomarker would be easily measurable in routine clinical laboratories making it cost-effective and accessible [8].

A paediatric study of children with bronchiectasis demonstrated that airway neutrophil count was higher in the presence of airway bacteria; however, 22% of children without lower airway infection had airway neutrophils above 85% [9]. Several studies investigated the airway microbiology in children with bronchiectasis [9,10]. The most common airway pathogens were found to be *Haemophilus influenzae*, *Streptococcus pneumoniae*, *Moraxella catarrhalis*, and less frequently *Pseudomonas aeruginosa* [11]. However, these studies were mostly in relatively small cohorts, and most only described the airway microbiology without exploring its association with airway neutrophilia or systemic inflammation.

Recently, predictive relationships between blood inflammatory indices to localized infection have been described [12]. An adult study post arthroscopy showed a predictive area under the curve (AUC) for diagnosing a periprosthetic joint infection with the monocyte/lymphocyte ratio (MLR) demonstrating an AUC of 0.79 (95% CI, 0.76–0.82) a neutrophil/lymphocyte ratio (NLR), with an AUC of 0.80 (95% CI, 0.78–0.82); the platelet/lymphocyte ratio (PLR) had an AUC of 0.84 (95% CI, 0.82–0.86) and a platelet/mean platelet volume ratio (PVR) with an AUC of 0.85 (95% CI, 0.81–0.89), in diagnosing periprosthetic joint infection [12]. Another potential marker of mean platelet volume (MPV) reflects platelet activation, and could be a biomarker for systemic inflammation [13]. In adult children with chronic obstructive pulmonary disease (COPD), NLR and PLR were increased significantly compared to controls and further increased during exacerbations [14].

Thus, in a large cohort of children with bronchiectasis we aimed to (i) describe airway pathogens (bacteria and viruses) and cytology in BAL, and (ii) evaluate if airway neutrophil percentage relates to potential peripheral blood markers of inflammation, specifically MLR, NLR, PLR and PVR, and CRP. We hypothesized that a peripheral inflammatory marker of airway neutrophilia can be defined in children with bronchiectasis.

## 2. Materials and Methods

We identified children who attended the Department of Respiratory and Sleep medicine at Queensland Children’s Hospital, diagnosed with bronchiectasis between February 2008 and July 2021, based on their clinical symptoms combined with a chest CT scan. Inclusion criteria were (i) presence of bronchiectasis on the chest CT scan (age < 18-years) using paediatric criteria of BAR of 0.8 [15], (ii) bronchoalveolar lavage (BAL) and full blood count undertaken within 3 months of diagnosis. Exclusion criteria were children with bronchiectasis due to cystic fibrosis (CF) or those with incomplete data. In our centre, we perform chest CT scans and BAL under the same anaesthetic (for children who are unable to get a chest CT scan while awake) while investigating for bronchiectasis. We avoid general anaesthetic in children if they are acutely unwell with fever or new respiratory symptoms. In cases of recent PCR positive respiratory viral infection or hospitalisation due to respiratory reasons, “elective anaesthesia” is deferred for ~4 weeks. In all children bronchoscopy and BAL were performed as part of the diagnostic evaluation of chronic wet cough and investigation for bronchiectasis.

Using a standardised data sheet, a single author (HW) extracted data from the patients’ electronic medical charts and pathology database. As data were already collected as part of clinical practice, informed consent was not obtained. The Children’s Health Queensland Human Research Ethics Committee approved the study (EX/2022/QCHQ/91831).

### 2.1. Data Collection

Demographic patient characteristics, blood, and BAL results were collected. These included haemoglobin, white cell count (WCC), platelets, monocytes, lymphocytes, neutrophils, basophils, eosinophils, and C-reactive protein (CRP). From these data, a priori defined inflammatory markers were calculated including the following:

(i) monocyte/lymphocyte ratio (MLR)
(ii) neutrophil/lymphocyte ratio (NLR)
(iii) platelet/lymphocyte ratio (PLR)
(iv) platelet/mean platelet volume ratio (PVR)

In our centre, all flexible bronchoscopy (Olympus; Tokyo, Japan) is performed under general anaesthesia and BAL is undertaken in accordance with the ERS guidelines. For lavage, sterile saline is instilled in three aliquots of 1 mL/kg (maximum 20 mL) into the right middle lobe and lingula in generalized disease and in the worst affected area in children with localized bronchiectasis. The first two aliquots pooled together are used for microbiology assessment, including aerobic bacterial cultures, testing for mycobacterial species and polymerase chain reaction (PCR) for mycoplasma and respiratory viruses. The third lavage aliquot is performed in the lobe where either the first or second lavage was performed. The third aliquot is used for cellular analysis, including total and differential cell counts. In the BAL cellular differential counts are expressed as percentages of total cell count, and positive bacterial culture is defined as ≥10^4^ colony-forming units (CFU)/mL.

We only included recognized respiratory pathogens, including *Streptococcus pneumoniae*, β-haemolytic streptococci, Haemophilus species, *Moraxella catarrhalis*, *S. aureus*, and Enterobacteria. Two bacteria, *Stenotrophomonas maltophilia* and *P. aeruginosa*, were considered an infection at a lower bacterial growth threshold (≥10^1^ cfu/mL). Respiratory infection was also considered to be present if any fungi or mycobacteria were isolated or viruses and mycoplasma (by PCR) were detected. We do not perform cycle threshold routinely after viral PCR testing. Respiratory viruses were classified as present/absent as detected by the polymerase chain reaction.

### 2.2. Statistical Analysis

Demographic data were expressed as percentages when categorical or, if continuous, as median (interquartile range [IQR]) as the data were not normally distributed. Non-parametric tests (Fisher’s exact test and Spearman rank correlation coefficient) were used throughout. To identify factors associated with airway neutrophilia(defined as BAL neutrophil > 15%) [16], we examined each factor individually using univariable logistic regression and those variables with *p*-values < 0.1 in a multivariable logistic regression model.

We used logistic regression to relate airway neutrophilia with peripheral blood markers of inflammation, i.e., MLR, NLR, PLR, PVR, and CRP, as well as to examine the relationship between the presence of bacteria or viruses and airway neutrophilia. We also used area under the curve receiver operating curves (ROC) to identify the discriminatory ability of each of these peripheral inflammatory markers to detect airway neutrophilia. A 2-tailed *p*-value of 0.05 was considered significant. All statistical analyses were performed with STATA version 17 (StataCorp, College Station, TX, USA).

## 3. Results

Of the 477 children, 75 children had incomplete data and were excluded, leaving 402 children in this retrospective cohort study (Table 1).

### 3.1. Population Characteristics

Out of 402 children included in this study, 172 (42.78%) were female. There were 67 (16.67%) children who identified as First Nations (Aboriginal, Torres Strait Islander, or both). The median age at which the children had a diagnostic chest CT-scan was 3.12 years (IQR 1.74–5.47) (Table 1).

### 3.2. Bronchoalveolar Lavage Cell Cytology

The results of BAL cytology are presented in Table 1. Airway neutrophilia (neutrophils > 15%) [16] was present in 279 children (69.40%) and airway eosinophilia (eosinophils > 1%) [17] was present in 77/395 children (19.49%).

### 3.3. Microbiology in BAL

Children numbering 355 (88.31%) had pathogenic bacteria cultured. *H. influenzae*, *α-Haemolytic streptococcus*, and *S. pneumoniae* were the most frequently cultured bacteria (Table 2). *P. aeruginosa* was identified in 4.48% of the BAL samples. A virus was positive on PCR in 131 children (32.58%) in the BAL. The most frequently identified virus was Adenovirus in 63 children (15.67%), followed by RSV in 19 (4.72%), and Rhinovirus in 14 (3.48%) children. Among the 131 children with BAL positive for a respiratory virus, two viruses were detected in 15 (3.73%) children, while 6 (1.49%) had three viruses present Bacteria-virus co-infection was identified in BAL in 117 children (29.10%). No bacteria or virus were identified in 31 (7.71%) children. Children numbering 240 (59.70%) had only bacteria identified, and 16 children (3.98%) had viruses only.

### 3.4. Bacteria and Airway Neutrophilia

Airway neutrophilia was present in 279 (69.40%) children. Children numbering 107 (26.62%) had bacteria cultured in the BAL in absence of airway neutrophilia, whereas airway neutrophilia was present in 31 (7.71%) participants without bacteria in the BAL.

We analysed eight bacterial species for their association with airway neutrophilia. We identified that airway neutrophilia was significantly associated with the presence of *H. influenzae* and *S. pneumoniae*; odds ratio (OR) 2.03 (95% CI 1.31–3.15, *p* = 0.002) and 2.41 (95% CI 1.36–4.29, *p* = 0.003), respectively. Combining both these in a multiple logistic regression resulted in OR 1.175 (95% CI 1.12—2.77, *p* = 0.015) for *H. influenzae* and an OR 2.03 (95% CI 1.13–3.69 *p* = 0.019) for *S. pneumoniae*. There was no significant assocaition of airway neutrophilia with the presence of other bacteria tested. Age at diagnostic chest CT scan was significantly asssociated with the presence of any bacteria in the BAL sample, a higher age in years was associated with decreased likelihood—OR 0.88 (95% CI 0.81–0.95, *p* = 0.001)—but there was no association between airway neutrophilia and the presence of any bacteria in the BAL.

### 3.5. Viruses and Airway Neutrophilia

Airway neutrophilia was associated with the presence of Adenovirus in BAL (OR 2.06 (95% CI 1.06–4.04, *p* = 0.033). No other virus was associated with airway neutrophilia. However, it must be noted that the PCR detection does not necessarily indicate active infection, and children could shed the virus after recovering from infection.

### 3.6. Peripheral Blood Values

Peripheral blood values, CRP and the calculated MLR, NLR, PLR, and PVR, are presented in Table 1. Logistic regression was used to investigate an association between airway neutrophilia (>15%) and MLR; however this was non-significant—OR 1.34 (95% CI 0.22–8.17, *p* = 0.75). Similarly there was no association between airway neutrophila and NLR with OR 1.03 (95% CI 0.90–1.18, *p* = 0.69), PLR with OR 1.00 (95% CI 0.99–1.00, *p* = 0.61), or PVR with OR 0.94 (95% CI 0.88–1.02, *p* = 0.15).

In the children where CRP was available (n = 203), raised CRP (>2.0 mg/L) was significantly associated with airway neutrophilia—OR 2.26 (95% CI 1.14–4.49, *p* = 0.019). Rising CRP was also associated with increasing airway neutrophil percentage (Spearman’s rho 0.28, *p* = 0.0001). CRP was missing in almost 50% children, randomly based on whether it was ordered on the day of bronchoscopy. We compared the characteristics of children with vs. without CRP (Table 3).

This comparison demonstrated that the age at chest CT scan (age at diagnosis of bronchiectasis) as well as blood lymphocyte count, platelet volume, MLR, NLR, PLR, and PVR were different between the two groups. We further examined the OR and ROC curve for the peripheral markers for identifying BAL neutrophilia and they remained insignificant for both groups.

The ROC-curves of various inflammatory marker ratios, with airway neutrophilia as univariable classifier, provided an AUC of 0.50 (95% CI 0.44–0.56) for MLR, AUC of 0.52 (95% CI 0.46–0.58) for NLR, AUC of 0.51 (95% CI 0.44–0.57) for PLR, AUC of 0.42 (95% CI 0.36–0.48) for PVR, and AUC of 0.57 (95% CI 0.50–0.65) for CRP.

While we performed a multiple logistic regression for the two bacteria found which were significantly associated with airway neutrophilia, we did not do this for the blood markers as none of the peripheral blood markers assessed were significantly associated with airway neutrophilia.

## 4. Discussion

We described the peripheral blood and BAL data for a large cohort of paediatric children with bronchiectasis. We found that airway neutrophilia was associated with the presence of *H. influenzae*; OR 2.03 (95% CI 1.31–3.15, *p* = 0.002) and the presence of *S. pneumoniae* 2.41 (95% CI 1.36–4.29, *p* = 0.003) in the BAL. We also found that the presence of Adenovirus in the BAL is significantly associated with airway neutrophilia; odds ratio 2.06 (95% CI 1.06–4.04, *p* = 0.033). Furthermore, airway neutrophilia was associated with raised blood CRP in a subgroup of children with bronchiectasis, but there was no association between airway neutrophilia and novel systemic inflammatory markers like MLR, NLR, PLR, and PVR.

Our results are similar to previous studies demonstrating that most common respiratory pathogens found in the BAL fluid were *H. influenzae*, *S. pneumoniae*, *M. catarrhalis*, and *S. aureus* [9,16]. *H. influenzae* is a common bacteria identified from the BAL of children with bronchiectasis [18]. However, an association between airway neutrophilia and *H. influenzae* has not been previously defined in children with bronchiectasis. A prospective study of a 5 year follow up of children with protracted bacterial bronchitis previously identified *H. influenzae* in BAL as an independent risk factor (OR (adj) = 5.1, 95% CI: 1.4–19.1) for developing bronchiectasis in children [19]. It is biologically plausible that *H. influenzae* leads to airway neutrophilia in these children. In addition, our study confirms that viruses are prevalent in a substantial number of children with bronchiectasis, even when not acutely unwell. No study previously reported associations with airway neutrophilia and airway viral infection.

The exact role of viruses in bronchiectasis has not been fully understood. Viruses are often identified during exacerbations in both children and adults [20,21,22,23]. At the same time, viruses can be identified during the steady state phase of bronchiectasis. A multicenter study in Italy reported the presence of at least one virus during the stable state in 12.8% of the adult children, regardless of time of year [24]. On the contrary, an Australian study with small numbers reported viruses to be present in 11 out of 12 (92%) of the stable children during winter and in 5/15 (33%) during the summer period [25]. In children 12% had a virus identified at the time of bronchoscopy in a small study [9]. Several studies reported viruses to be associated with pulmonary exacerbations, *Rhinovirus* being the most common [23]. We identified viruses in BAL from 32.58% of the children, which is substantially higher compared to other non-Australian studies, both in adults and children. Since our cohort consists of children diagnosed throughout the year over 15 years, the time of the year most likely does not influence the findings, whereas some studies reported significant differences dependant on the time of year. This might partially be explained by geographical differences among different study locations. Additionally, the virus detection threshold of the PCR analyses and time of year might influence the findings. Furthermore, our data are from BAL samples, potentially improving the PCR detection rate.

Indigenous ethnicity was previously described as associated with high TCC in BAL [16], but we did not see that association in our study. This could be because the earlier study had children with protracted bacterial bronchitis, chronic suppurative lung disease, and bronchiectasis pooled together, and their patient population was 45% Indigenous Australians compared to 16% in our study. Lower age has been associated with increased incidence of lower respiratory tract infection in children with bronchiectasis [26]. Our study showed similar findings. Age at time of diagnosis was significantly associated with the presence of bacteria; odds ratio 0.88 (95% CI 0.81–0.95, *p* = 0.001).

Our hypothesis was that peripheral inflammatory markers to identify airway neutrophilia can be identified using derivatives from peripheral blood in children with bronchiectasis. Neutrophil to lymphocyte ratio values were previously shown to be statistically greater in adults with bronchiectasis exacerbation compared to healthy controls [27].

In adults, the bacterial load in sputum has been associated with the markers of airway inflammation measured in sputum. Compared with the 22.1% of adult children whose sputum cultures failed to grow potentially pathogenic microorganisms, significantly higher levels of inflammatory markers were found when the bacterial colony count reached 10^5^ colony-forming units per mL [28]. The median levels of each of the inflammatory markers increased progressively with increasingly bacterial load and decreased after 14-days of intravenous antibiotics as the bacterial load declined in sputum [28].

In a study of 50 children with bronchiectasis, the average blood leukocyte count (*p* < 0.001), platelet count (*p* = 0.018), absolute neutrophil count (*p* < 0.001), and NLR (*p* < 0.001) were higher, compared with the control group [29]. NLR values, in the period of acute exacerbation were significantly higher than the values of both the non-exacerbation periods (*p* = 0.02) and the control group (*p* < 0.001). In contrast, MPV values in the period of acute exacerbation did not exhibit a significant difference from those of non-exacerbation periods (*p* = 0.530) and the control group (*p* = 0.103) [29]. Our data also support these results that NLR and MPV are not raised during the stable state in children with bronchiectasis.

On the contrary, in a study in adults with bronchiectasis, investigating NLR and outcomes of severity in bronchiectasis during stable state and exacerbation, NLR proved to have a significant correlation with FACED [30] (Forced expiratory volume in 1 s, Age, Chronic colonization, Extension, and Dyspnea), E-FACED (exacerbation-FACED) [31], and BSI scores (Bronchiectasis severity index) [32]. Additionally, higher NLR was associated with lower quality of life, more comorbidities, more pathogenic micro-organisms, and applied treatment. They suggested NLR as a practical biomarker for exacerbation severity and for predicting new exacerbations in adults [32]. We could not find an association between airway neutrophilia and NLR in children during bronchoscopy at the time of diagnosis, likely because they were in a stable state.

An adult study investigated the correlation between two clinical scoring systems: FACED and BSI and the presence of systemic inflammation, in adult children with stable bronchiectasis. CRP levels, leukocyte count, and NLR were compared to the FACED and BSI score. Only CRP proved to have a significant correlation to both scoring systems in the steady state phase, identifying CRP as a useful biomarker for disease severity [33]. Another adult study identified raised CRP as a risk factor for lung function decline; (OR 3.1, 95% CI = 1.88–8.91 *p* = 0.023) [34]. Furthermore, an adult study demonstrated that high sensitivity-CRP level at stable state can predict bronchiectasis exacerbation in the subsequent year [35]. Similarly, our study found CRP to be associated with airway neutrophilia in children with bronchiectasis during stable state. CRP is an acute phase reactant and can rise with infection as well as inflammation. Even though we demonstrated an association between raised CRP and airway neutrophilia, this is not specific to bronchiectasis and hence should be interpreted with caution.

A possible explanation as to why no association could be found between peripheral inflammatory markers (except CRP) and airway neutrophilia might be that BALs were performed during a presumed stable/steady state of bronchiectasis. Even though some of these children would have had chronic wet cough, it is common practice to avoid general anaesthesia in children when they are acutely unwell, therefore the majority of diagnostic bronchoscopies in our cohort were likely performed during the stable state. Even though, airway neutrophilia can be present during the steady state phase, peripheral blood and BAL results likely differ from that seen during an exacerbation. Peripheral blood neutrophil count and CRP were shown previously to be increased in children with bronchiectasis during exacerbation [26]. Similarly, peripheral neutrophil count can be normal during the steady state and increase during exacerbations [36]. Therefore, our study might not appropriately reflect a relationship between airway neutrophilia and peripheral inflammation markers during exacerbations.

Our study has many strengths. First, we described the airway microbiology and cytology in a large cohort of children with bronchiectasis. To our knowledge, this is the largest cohort of children with bronchiectasis with BAL and peripheral blood results. Second, we selected these children from a large database over many years removing the selection bias.

Our study is retrospective and hence has several limitations that include the lack of a control group and incomplete capture of all data. Second, BAL is generally performed under general anaesthesia, therefore we assumed that BAL was performed in a steady state phase. It is possible that some children had an unrecognised exacerbation at the time of bronchoscopy, leading to higher pathogen detection, raised BAL TCC, and increased airway neutrophilia, although it did not lead to raised inflammatory markers. We also did not collect data about the use of acute or prophylactic antibiotics which could affect the airway microbiology and airway cellularity. We only used data from the first bronchoscopy performed around the time of diagnosis of bronchiectasis; therefore, it is less likely that macrolide usage will be a major confounding factor here, as children are often started on prophylactic antibiotics after a diagnosis of bronchiectasis, although the use of an acute course of antibiotics could still affect the BAL results. We acknowledge that we do not have objective information about the clinical state of the child compared to their well baseline/stable state. Lastly, we did not collect data on aetiology of bronchiectasis as it is biologically plausible that systemic inflammation and airway neutrophilia may vary across different etiologies. Adult data have shown that bronchiectasis and airway damage can progress independently of the inciting etiology [37].

In conclusion, our study did not identify a peripheral blood biomarker for airway neutrophilia. There was an association between raised CRP and airway neutrophilia in a subgroup of children, but it is not specific. Since newer anti-neutrophilic/anti-inflammatory treatments [38,39] are becoming available for children with bronchiectasis, a reliable non-invasive biomarker for bronchiectasis in children will help in identifying potential targets for choosing children for treatment and monitoring response. A reliable lower airway sample is difficult to obtain in children who cannot expectorate, therefore using innovative approaches to identify new non-invasive biomarkers in children is needed [8]. Examples of minimally invasive biological samples for children include urine, exhaled breath condensate, saliva, and peripheral blood. A prospective study with additional information including disease severity, exacerbation or stable phase, disease duration, and prophylactic antibiotic use to investigate the role of the peripheral blood markers discussed in this study could provide further insight into this. We also need to identify non-invasive biomarkers to identify children with chronic cough at risk of developing bronchiectasis and identify exacerbations early to be able to introduce in time intervention.

## Figures and Tables

**Table 1:** Characteristics of children included in the study.

Characteristic	N = 402 (Median (IQR) or n, %)
Age at chest CT scan	3.12 (1.74–5.47)
First Nations	67 (16.7%)
Male gender	231 (57.5%)
Blood neutrophils (×10^9^)	3.37 (2.3–4.93)
Blood lymphocytes (×10^9^)	4.47 (3.26–5.94)
Blood eosinophils (×10^9^)	0.33 (0.16–0.63)
Platelet count (n = 396)	331.5 (276.5–401)
Platelet volume	9.5 (9–10.2) (n = 400)
MLR *	0.16 (0.12–0.23)
NLR ^†^	0.735 (0.48–1.21)
PLR ^‡^	74.29 (56.14–97.06)
CRP ^§^	1.9 (1.9–3.7) (n = 203)
BAL total cell count	350 (130–950) (n = 390)
BAL neutrophils (%)	41 (11–75.9) (n = 394)
BAL eosinophils (%)	0 (0–0.7) (n = 395)
BAL lymphocytes (%)	9.00 (4.2–17.00) (n = 398)
BAL macrophages (%)	42 (13–70.2) (n = 395)
Bacterial growth in BAL	355 (88.3%)
Viral species identified in BAL	131 (32.54%)

Data are presented as n (%) or median (IQR). CT = computed tomography; * MLR = monocyte/lymphocyte ratio; **^†^** NLR = neutrophil/lymphocyte ratio; **^‡^** PLR = platelet/lymphocyte ratio. **^§^** CRP = C-reactive protein, BAL = bronchoalveolar lavage.

**Table 2:** Frequency of eight clinically significant bacteria present in BAL.

Bacteria	Frequency ^A^ (% ^B^)
*Haemophilus influenzae*	185 (46.02)
*α-Haemolytic streptococcus*	169 (42.04)
*Streptococcus pneumoniae*	95 (23.63)
*Moraxella catarrhalis*	56 (13.93)
*Staphylococcus aureus*	27 (6.72)
*Pseudomonas aeruginosa* ^C^	18 (4.48)
*β-Haemolytic streptococcus*	8 (1.99)

^A^ Frequencies are based on a cut off > 104 cfu/mL, where lower than the threshold is considered as non-significant bacteria being present. ^B^ Percentage of total study sample; some children had more than one bacterium identified. ^C^ For Pseudomonas aeruginosa, a lower threshold of 101 cfu/mL was used.

**Table 3:** Comparison of children with and without CRP data available.

	CRP Not Available n = 199	CRP Available n = 203	*p*-Value
Age at chest CT scan	3.92 (2.1–6.25)	2.52 (1.51–4.48)	<0.001
First Nations	29 (14.6%)	38 (18.7%)	0.26
Male gender	112 (56.3%)	119 (58.6%)	0.64
Blood neutrophils (×10^9^)	3.5 (2.23–5.13)	3.27 (2.32–4.78)	0.75
Blood lymphocytes (×10^9^)	3.97 (3–5.28)	5.02 (3.65–6.68)	<0.001
Blood monophils (×10^9^)	0.74 (0.54–0.94)	0.75 (0.59–0.96)	0.19
Blood eosinophils (×10^9^)	0.33 (0.16–0.67)	0.33 (0.16–0.59)	0.49
Platelet volume	9.6 (9.1–10.3)	9.4 (9–10)	0.02
MLR	0.18 (0.13–0.24)	0.15 (0.11–0.22)	<0.001
NLR	0.82 (0.53–1.31)	0.65 (0.43–1.09)	0.003
PLR	77.31 (57.91–104.59)	69.25 (55.18–90.15)	0.008
PVR	2.43 (1.78–3.21)	1.89 (1.39–2.58)	<0.001
BAL cell count	340 (110–830)	360 (140–980)	0.35
BAL macrophages	39 (12.2–71)	43.85 (14.3–70)	0.58
BAL lymphocytes	7.6 (4.2–16.8)	10 (4.5–17.5)	0.35
BAL neutrophils	40 (12–77)	41.8 (10.8–74.8)	0.75
BAL eosinophils	0 (0–0.5)	0 (0–0.8)	0.047
Virus present	63 (31.7%)	70 (34.5%)	0.55
Bacteria present	170 (85.4%)	185 (91.1%)	0.075

Data are presented as n (%) or median (IQR). CT = computed tomography; MLR = monocyte/lymphocyte ratio; NLR = neutrophil/lymphocyte ratio; PLR = platelet/lymphocyte ratio. CRP = C-reactive protein, BAL = bronchoalveolar lavage.

## Data Availability

The original contributions presented in this study are included in the article. Further inquiries can be directed to the corresponding author.
